# Frequency of cigarette smoking and its associated factors among men in East Africa: a pooled prevalence analysis of national survey using multinomial regression

**DOI:** 10.1186/s12889-024-18188-4

**Published:** 2024-03-01

**Authors:** Bewuketu Terefe, Mahlet Moges Jembere, Bogale Chekole, Nega Tezera Assimamaw, Daniel Ayelegne Gebeyehu

**Affiliations:** 1 Department of Community Health Nursing, School of Nursing, College of Medicine and Health Sciences, University of Gondar, Gondar, Post Office Box: 196, Ethiopia; 2 Department of Emergency and Critical Care Nursing, School of Nursing, College of Medicine and Health Sciences, University of Gondar, Gondar, Ethiopia; 3 Department of Comprehensive Nursing, College of Medicine and Health Sciences, Wolkite University, Southern, Ethiopia; 4 Department of Pediatrics and Child Health Nursing, School of Nursing, College of Medicine and Health Sciences, University of Gondar, Gondar, Ethiopia; 5 Department of Psychiatry, School of Medicine, College of Medicine and Health Sciences, University of Gondar, Gondar, Ethiopia

**Keywords:** Frequency, Cigarette smoking, Factors, Men, East Africa, Multinomial regression

## Abstract

**Background:**

Despite the harmful effects of smoking, there have been few studies to pinpoint the factors of this habit, and little is known about it in the East African region. For this reason, this study sought to determine the frequency and factors of cigarette smoking among men in the region.

**Methods:**

Data from recent demographic and health surveys carried out in ten East African countries between 2015 and 2022 were analyzed in this study. Data from 87,022 men was collected. The key factors affecting the smoking rates in the area were investigated using binary and multiple multinomial logistic regression. To ascertain if variables were statistically significant in the final model for binary regression and multiple regression, *P* values of ≤ 0.2 and < 0.05 were used respectively.

**Results:**

Overall, about 14.69% of people currently smoke cigarettes. Of this about 11.03 (95% CI = 10.82, 11.24) was for daily active tobacco use. As compared to < 26-year-old men, men with an age range of 26–35 years (RRR = 2.17, 95% CI: 2.01,2.34), 36–45 years (RRR = 2.82, 95% CI: 2.60, 3.07), and > 45 years old (RRR = 3.68, 95% CI: 3.38, 4.02), were using cigarettes daily rather than no-smoking cigarettes. Men who had begun their first sexual intercourse at the age of 7–19 years (RRR = 6.27,95% CI, 5.35,7.35), 20–25 years (RRR = 4.01, 95% CI, 3.40,4.72), and greater than 25 years old (RRR = 3.08, 95% CI, 2.55,3.71) have shown a higher relative risk ratio to smoke cigarette daily rather than using not smoke cigarette respectively, married (RRR = 0.86, 95% CI, 0.79,0.93), divorced or widowed (RRR = 2.51, 95% CI, 2.27,2.77), middle wealth index (RRR = 2.11, 95% CI 1.98,2.24), and rich (RRR = 1.44, 95% CI, 1.34,1.54), secondary/higher education (RRR = 0.72, 05% CI, 0.66,0.77), rural men (RRR = 0.69, 95% CI, 0.65,0.73), employed men (RRR = 1.26,95% CI, 1.17,1.36), mass media exposure (RRR = 0.76, 95% CI, 0.73,0.81), men who have one sex partner (RRR = 1.23,95% CI,1.13,1.35), and more than one sex partner (RRR = 1.63, 95% CI, 1.47,1.79) more times as compared to those participants who had no sex partner respectively.

**Conclusions:**

Men in East African nations were substantially more likely to smoke cigarettes if they were older, had less education, had a higher wealth index, were divorced or widowed, had many sexual relationships, had early sexual activity, resided in an urban area, were employed, or had no media exposure. The identified factors should be considered by policymakers and public health professionals to lower smoking initiation and increase smoking cessation among men.

## Introduction

The use of tobacco is one of the major preventable causes of death worldwide, contributing to an epidemic of Non-Communicable Diseases (NCDs) such as cancer, heart disease, stroke, chronic lung disease, and others [1]. Additionally, it is a significant global preventable cause of early death and disease [1, 2]. Around 1.4 billion adults worldwide, comprising 1.07 billion smokers and 367 million smokeless tobacco users, used tobacco in 2017; the age-standardized global average prevalence of smoking is 19.2% [3]. It led to almost 8 million deaths annually, with those over 45 experiencing a markedly higher mortality risk [3, 4].

According to studies conducted in wealthy nations, the rate of cigarette smoking has sharply declined recently [5]. However, it is frighteningly rising in low-income nations [6, 7]. According to the WHO estimate for 2023, 80% of the 1.3 billion tobacco smokers worldwide reside in low- and middle-income nations [1]. About 36.7% of males and 7.8% of women worldwide used cigarettes in 2020, totaling 22.3% of the population [1]. According to data from the Global Tobacco Surveillance, men in sub-Saharan Africa smoke cigarettes at rates that range from 20 to 60% nationally, and both men and women are using tobacco more often each year [8]. Men and women smoke at rates of 4% and less than 1%, respectively, per the 2016 Ethiopian Demographic and Health Survey (EDHS) report [9].

Numerous studies on the effects of smoking cigarettes have been done. Cigarette smoking among men is attributed to a number of factors, including exposure to smokers (friends, parents, and teachers), the availability of tobacco, low socioeconomic status, subpar academic performance, low self-esteem, a lack of perceived risk of use, and a lack of ability to fend off influences to use tobacco [10–12]. Additionally, marital status, wealth, occupation, education, engaging in physical altercations, abusing alcohol, using marijuana, and engaging in sexual activity are all linked to smoking cigarettes [13–15].

The WHO set a goal to reduce mortality from chronic respiratory illnesses, cancer, diabetes, and cardiovascular diseases by 25% among people aged 30 to 70 between 2010 and 2025 [16]. The most efficient and cost-effective way to prevent non-communicable diseases in East African nations is to reduce cigarette consumption. Assessing the prevalence and related characteristics in East African nations is crucial because men smoke more cigarettes than smokeless tobacco. Several African nations have ratified the WHO Framework Convention on Tobacco Control, which forbids the use of cigarettes and any other tobacco products in any indoor or outdoor space that may influence child tobacco use [17, 18]. However, there is a lack of information on smoking in East Africa. Furthermore, gender disparity (males smoke more frequently than females), recent empirical knowledge, and current evidence gaps using the recent national health survey data were among the top research gaps that have been addressed in this study. Therefore, the purpose of this study was to utilize multinomial logistic regression to determine the prevalence, frequency, and associated characteristics of cigarette smoking among men in East Africa.

## Methods

### Study setting and period

With the most recent Demographic and Health Survey (DHS) data, we conducted secondary data analysis in the following East African nations from 2011 to 2022: Burundi, Ethiopia, Kenya, Madagascar, Malawi, Rwanda, Tanzania, Uganda, Zambia, and Zimbabwe. Sample size, and study periods are described below (Table 1).

**Table 1:** Countries, sample size, and survey year of demographic and health surveys included in the analysis for 10 East African countries

Country	Survey year	Sample size(weighted)	Frequency(weighted)
Burundi	2016/17	7,552	8.68
Ethiopia	2016	12,688	14.58
Kenya	2022	14,453	16.61
Madagascar	2021	9,037	10.38
Malawi	2016/17	7,478	8.59
Rwanda	2015/16	6,513	7.48
Tanzania	2015	3,514	4.04
Uganda	2016	5,336	6.13
Zambia	2018	12,132	13.94
Zimbabwe	2015	8,319	9.56

With a significant emphasis on indicators of fertility, reproductive health, mother and child health, mortality, nutrition, and self-reported health behaviors among adults, the DHS gathers a wide range of objective and self-reported data. Epidemiological research that monitors prevalence, trends, and inequities is made easier by data from the DHS. For the population of the nation, it drew samples that were nationally representative. The nature of the datasets from demographic and health surveys has been described in full elsewhere [19].

### Data source

The demography and health survey (DHS) program’s official database, www.measuredhs.com, was used to obtain the data. Population, health, and nutrition monitoring and impact evaluation indicators can be used using data from demographic and health surveys, which are nationally representative household surveys. The DHS employs a stratified two-stage cluster design, with enumeration areas (EA) being the first stage and a sample of homes being picked from each EA in the second. Comprehensive survey methodology [19].

### Sample size determination and sampling method

For around 10 of the 13 countries in East Africa, there were recent Demographic and Health Survey (DHS) reports available. The most current conventional census frame was used for each survey taken in the nations mentioned below. The DHS samples frequently divide each administrative geographic region into urban and rural areas. During the first round of sampling, enumeration areas (EAs) were chosen with a probability proportional to their size within each stratum. The systematic sampling strategy chooses a specified number of homes in the chosen EAs in the second stage. After listing the households, a certain number of households from the designated cluster are chosen using equal probability systematic sampling [19].

### Data management and statistical analysis

The data set was collected from the website https://dhsprogram.com after receiving a letter from the DHS approving its utilization. Data extraction, recoding, and analysis are performed using Stata version 17. The study included weighting to ensure representativeness, reduce the non-response rate, and produce an accurate statistical estimate (robust standard error) [20]. In binary regression, those variables whose values were less than or equal to 0.20 were selected as candidate variables for the multiple multinomial logistic regression model to estimate factors associated with smoking at a 95% confidence interval (Table 2). In the binary regression, each independent variable was regressed independently to assess whether it fits the minimum *p*-value of less than or equal to be selected as a candidate variable for the final model. To identify the contributing factors to the prevalence of cigarette smoking among men, a multiple multinomial logistic model was used. The model’s best fit Adjusted Odds Ratio (AOR) with 95% CI was supplied. The descriptive data were compiled using descriptive research, such as frequency count and proportion for categorical data. Bivariable logistic regression was used to choose potential variables for multiple multinomial logistic regression. A logistic model was fitted to test for multicollinearity among the independent variables using the variance inflation factor. The overall fitness of the final regression model was further assessed using the Hosmer and Lemeshow test. The statistical significance level for the final model was set at < 0.05.

**Table 2:** Crude Relative risk ratio on the binary multinomial logistic regression among men in East Africa

Smoking frequencies: do not smoke taken as outcome (reference group)	Crude relative risk ratio, 95% confidence interval			
	Everyday	*P* value	Somedays	*P* value
Age in years	1.70 (1.67,1.73)	0.0001	1.31(1.27,1.35)	0.0001
Age at first sex	1.41(1.37,1.45)	0.0001	1.38(1.32,1.44)	0.0001
Marital status	2.17(2.11,2.23)	0.0001	1.80(1.72,1.88)	0.0001
Wealth index	0.68(0.67,0.70)	0.0001	0.82(0.78,0.85)	0.0001
Education status	0.62(0.61,0.64)	0.0001	0.93(0.88,0.98)	0.006
Residence	1.23(1.18,1.29)	0.0001	0.93(0.86,0.99)	0.005
Currently working	2.76(2.57,2.96)	0.0001	1.82(1.64,2.02)	0.0001
Mass media exposure	0.67(0.64,0.70)	0.0001	0.87(0.81,0.93)	0.0001
Number of sex partners	1.99(1.93,2.06)	0.0001	1.67(1.58,1.77)	0.0001
Sex of the household head	0.67(0.63,0.71)	0.23	0.88(0.79,0.97)	0.23
Use of internet	0.49(0.46,0.51)	0.22	0.83(0.77,0.89)	0.21

### Model goodness fits and comparison

As candidate models, three models binary logistic, ordinal, and multinomial logistic regressions were chosen. Given that smoking rates were listed as (no smoking, every day, and occasionally), an approach that was widely utilized was the ordinal logistic regression mode. However, after we tested the proportional odds model (POM) for model Stata command or Brant test, the assumption of the POM was not met, therefore we used a multinomial logistic regression model instead. Actually, there were modest differences between the three models and important parameters. To choose the best ordinal model for the data, use the POM assumptions, which state that all independent factors’ effects are constant across all categories of the outcome variable. The Brant test (*p* = 0.0001) and the omodel Stata tool were used to confirm the POM assumption’s validity. Comparing ordinal logistic regression models to multinomial models, the AIC, BIC, and LLR were similarly slightly bigger in the ordinal models. As a result, we did not use the POM to examine the relationships between the independent variables and the three categories of cigarette smoking frequencies among men. Therefore, compared to binary and ordinal logistic regressions, the multinomial logistic model was determined to be the best fitting model.

### Variance inflation factor (VIF) analysis

Prior to moving on to the analysis phase, each dependent variable’s variance inflation factors and tolerances were evaluated (the specifics are covered in the methodology section). This study’s overall mean VIF was 1.09.

### Variables of the study

Dependent variable: the outcome variable of this study was Frequency currently smokes tobacco among men the frequency of currently smoking was given as no smokes (recoded as “0”, every day (recode as “1”), and some days (recoded as “2”). Outcome ascertainments, and data management was done according to the Guide to DHS statistics [19]. After reviewer previous literatures on independent variables regarding smoking the following independent variables included age, age at first sex, number of sex partners, marital status, education, place of residence, wealth index, mass media exposure, currently working status, internet utilization, and sex of the household head were considered [11, 12, 14, 21, 22]. Based on the guide to DHS statistics missing data on whether each type of tobacco was smoked is assumed to indicate non-use of that specific type of tobacco. These missing data are excluded from the numerator but included in the denominator [19].

## Results

### Sociodemographic characteristics of the study participants

This survey comprised weighted samples from 87,022 men in 10 East African countries. In terms of age, approximately 36,841 (42.34%) of the study participants were just below 26 years old. Regarding place of residence and marital status, 62,462 (71.78%) and 35,615 (40.93%) lived in rural areas and were married, respectively. In terms of educational level, 38,679 (44.45%) of the participants had completed primary school. Media exposure (at least one media exposure among watching television, listening to radio, or reading newspapers or books) was reported by 55,517 (63.80%) of the survey participants. The majority of 73,960 (84.99%) households are headed by men. More than half (62,611) (71.95%) of them did not use the internet in the past 12 months. Slightly more than half (49,578; 56.97%) of the study participants have only one sex partner. About 45,115 (51.84%) of them had started their first sex exposure during their teenage years. Finally, about 39,945 (45.90%) of men came from rich households (Table 3).

**Table 3:** Sociodemographic, and other variables characteristics of smoking frequencies among men in East Africa

Smoking frequencies	Weighted sample	Weighted frequency
Age in years		
< 26	36,841	42.34
26–35	22,537	25.90
36–45	16,653	19.14
> 45	10,992	12.63
Age at first sex		
Never	17,530	20.14
7–19	45,115	51.84
20–25	20,172	23.18
> 25	4,205	4.83
Marital status		
Never married	35,615	40.93
Married	47,681	54.79
Widowed/ divorced	3,727	4.28
Wealth index		
Poor	29,824	34.27
Middle	17,253	19.83
Rich	39,945	45.90
Education status		
Not educated	9,714	11.16
Primary	38,679	44.45
Secondary/higher	38,629	44.39
Residence		
Urban	24,560	28.22
Rural	62,462	71.78
Currently working		
No	15,235	17.50
Yes	71,786	82.50
Mass media exposure		
No	31,505	36.20
Yes	55,517	63.80
Number of sex partners		
No	23,646	27.17
One	49,578	56.97
> 1	13,799	15.86
Sex of the household head		
Male	73,960	84.99
Female	13,062	15.01
Use of internet		
Never	62,611	71.95
Yes (last 12 months)	22,420	25.76
Yes (before 12 months)	1,991	2.29

### Factors associated with smoking among East African men

When all other variables were held constant, the variables age (26–35, 36–45, and > 45 years old) were associated with a 2.17, 2.82, and 3.68 times increase in the relative risk ratio (RRR = 2.17, 95% CI, 2.01,2.34), (RRR = 2.82, 95% CI, 2.60, 3.07), and (RRR = 3.68, 95% CI, 3.38, 4.02), respectively, of using a cigarette daily rather than no cigarette when compared to less than 26-year-old men, respectively. Similarly, as compared to men who had no sextual intercourse, those men who had begun their first sexual intercourse at the age of 7–19 years, 20–25 years, and greater than 25 years old have shown a higher relative risk ratio of (RRR = 6.27, 95% CI, 5.35–7.35), (RRR = 4.01, 95% CI, 3.40–4.72), and (RRR = 3.08, 95% CI, 2.55–3.71) more times to smoke cigarettes daily than those who did not smoke cigarettes, respectively. Furthermore, when all other variables were held constant, comparing with single men, those men who had married (RR = 0.86, 95% CI, 0.79, 0.93), and who had divorced or widowed (RR = 2.51, 95% CI, 2.27, 2.77), were times lower to smoke cigarettes daily than to not smoke cigarettes at all, respectively. Regarding wealth index, men with middle and rich households have shown a higher (RRR = 2.11, 95% CI: 1.98–2.24) and (RRR = 1.44, 95% CI: 1.34–1.54) relative risk ratio to smoke daily rather than not smoking cigarettes at all, respectively. However, men who had completed secondary or high education levels had shown a lower relative risk (RR = 0.72, 05% CI, 0.66–0.77) of smoking daily than uneducated men. Similarly, rural men revealed a lower risk ratio of being daily smokers as compared to urban men (RR = 0.69, 95% CI, 0.65–0.73). Conversely, when all other variables were held constant, employed men had shown a higher risk ratio (RR = 1.26, 95% CI, 1.17, 1.36) to utilize cigarettes daily rather than not smoking them at all. Likely, men who had mass media exposure about cigarette smoking had shown a lower relative risk ratio of being a daily smoker (RR = 0.76, 95% CI, 0.73–0.81) as compared to unexposed individuals. Men who have one and more than one sex partner had a higher tendency to smoke cigarettes daily rather than not be smokers at all, with a relative risk ratio of (RRR = 1.23, 95% CI: 1.13–1.5) and (RRR = 1.63, 95% CI: 1.47–1.79) more times as compared to those participants who had no sex partner, respectively (Table 4).

**Table 4:** Multiple multinomial logistic regression analysis results on associated factors of cigarette smoking frequencies among men in East Africa

Smoking frequencies in three categories (no smoking taken as a base outcome)							
Smoking frequencies	No smoking, n (%)	Every day, n (%)	Someday, n (%)	Std. errs.	z	*P* > z	Adjusted RRR (95% CI)
Age							
< 26	34,249(92.97)	1,700(4.61)	892(2.42)				1
26–35	18,583(82.46)	2,861(12.70)	1,092(4.85)	0.08	19.77	0.0001	**2.17(2.01,2.34)**
36–45	13,145(78.93)	2,767(16.61)	742(4.45)	0.12	24.55	0.0001	**2.82(2.60,3.07)**
> 45	8,262(75.16)	2,27(20.67)	458(4.17)	0.16	29.28	0.0001	**3.68(3.38,4.02)**
Age at first sex							
Never	17,208(98.16)	180(1.03)	143(0.81)				1
7–19	36,240(80.33)	6,781(15.03)	2,093(4.64)	0.51	22.66	0.0001	**6.27(5.35,7.35)**
20–25	17,148(85.01)	2,217(10.99)	807(4.00)	0.34	16.52	0.0001	**4.01(3.40,4.72)**
> 25	3,643(86.63)	421(10.01)	142(3.36)	0.29	11.81	0.0001	**3.08(2.55,3.71)**
Marital status							
Never married	33,088(92.90)	1,659(4.66)	868(2.44)				1
Married	38,879(81.54)	6,797(14.26)	2,004(4.20)	0.04	-3.74	0.0001	**0.86(0.79,0.93)**
Widowed/ divorced	2,272(60.97)	1,143(30.67)	312 (8.36)	0.13	17.86	0.0001	**2.51(2.27,2.77)**
Wealth index							
Poor	23,930(80.24)	4,571(15.32)	1,324(4.44)	0.07	22.97	0.0001	**2.11(1.98,2.24)**
Middle	14,840(86.02)	1,802(10.44)	611(3.54)	0.05	10.09	0.0001	**1.44(1.34,1.54)**
Rich	35,469(88.79)	3,227(8.08)	1,249(3.03)				1
Education status							
Not educated	7,771(80.00)	1,600(16.47)	343(3.53)				1
Primary	32,288(83.48)	4,933(12.75)	1,458(3.77)	0.03	-1.63	0.102	0.95(0.89,1.01)
Secondary/higher	34,180(88.48)	3,066(7.94)	1,383 (3.58)	0.03	-8.92	0.0001	**0.72(0.66,0.77)**
Residence							
Urban	21,184(86.25)	2,400(9.77)	977(3.98)				1
Rural	53,055(84.94)	7,200(11.53)	2,207(3.53)	0.02	-11.88	0.0001	**0.69(0.65,0.73)**
Currently working							
No	14,102(92.59)	753(4.94)	376(2.47)				1
Yes	60,129(83.76)	8,846(12.32)	2,808(3.91)	0.05	5.75	0.0001	**1.26(1.17,1.36)**
Mass media exposure							
No	26,158(83.03)	4,139(13.14)	1,208(3.83)				1
Yes	48,081(86.61)	5,461(9.84)	1,976(3.56)	0.02	-10.87	0.0001	**0.76(0.73,0.81)**
Number of sex partners							
No	22,345(94.50)	889(3.76)	412(1.74)				**1**
One	40,991(82.68)	6,443(13.00)	2,143(4.32)	0.06	4.51	0.0001	**1.23(1.13,1.35)**
> 1	10,903(79.01)	2,267(16.43)	629(4.56)	0.08	9.79	0.0001	**1.63(1.47,1.79)**
_cons				0.01	-52.70	0.0001	0.01(0.01,0.02)

Where bold confidence intervals are significant at *p* < 0.05

## Discussion

Our results show that among the study’s male participants, the frequency of current daily tobacco use was 11.03 (95% CI = 10.82, 11.24) and the frequency of some days usage was 3.66 (95% CI = 3.54, 3.79). Overall, about 14.69% of people currently smoke cigarettes. Our analysis revealed that significant risk factors for cigarette smoking among men in East African countries included higher age, lower education, wealth index, marital status, early sexual activity, multiple sexual partners, media exposure, living in a rural area, and employment status.

When all other factors were held constant, the age ranges of 26 to 35, 36 to 45, and > 45 years old had strong statistical associations with the likelihood of smoking cigarettes daily as opposed to abstaining from smoking (RRR = 2.17, 95% CI, 2.01,2.34), 2.82 to 3.07, and 3.68 to 4.02 as compared to men under the age of 26. This result was consistent with several research conducted in various fields, as older men were found to smoke more frequently than younger males in Ghana [23], Ethiopia [15], five South Asian nations [24], and India [25]. This may have more to do with delaying the start of tobacco use than it does with preventing people from starting to smoke [26]. Why older men smoke more than younger men may also be influenced by social and demographic factors, as well as nicotine dependence. Another study discovered that men who fall into lower socioeconomic and demographic groups (older, uneducated, and poor) are more likely to smoke. Younger males (15–29 years old and 30–44 years old) are discovered to have a decreased risk of smoking compared to older men (45–59 years old). Furthermore, it has been noted that males in higher wealth groups are less likely to smoke than their counterparts in lower wealth categories [15, 23]. However, in terms of smoking patterns, the American Lung Association reports that among men aged 26 or older, heavy smoking rates (more than 24 cigarettes per day) declined by 20% from 1974 to 2018. This shows that the number of elderly men who smoke has been declining over time [27]. Therefore, more research is required to uncover the true causes of age-related triggers in men.

This study discovered that, when all other variables were held constant, comparing with single men, those men who had married (RRR = 0.86, 95% CI, 0.79,0.93), and who had divorced or widowed (RRR = 2.51, 95% CI, 2.27,2.77), times lower to smoke cigarette daily rather than not smoking cigarette at all respectively. Significant psychological suffering can ensue from a widowed, divorce or marital separation, which frequently leads to smoking as a coping mechanism [28, 29]. According to the centers for disease control and prevention (CDC), current cigarette smoking was highest among Americans who were divorced, separated from their partners, or widowed and lowest among those who were married or cohabitating [30].

Men who have one, and more than one sex partners had a higher tendency of smoking cigarette daily rather than to be not a smoker at all with a relative risk ratio of (RRR = 1.23,95% CI,1.13,1.35), and (RRR = 1.63, 95% CI, 1.47,1.79) more times as compared to those participants who had no sex any partner respectively. Men who had their first sextual encounters between the ages of 7 and 19 years, 20 and 25 years, and older than 25 years showed a higher relative risk ratio of (RRR = 6.27, 95% CI, 5.35,7.35), (RRR = 4.01, 95% CI, 3.40,4.72), and (RRR = 3.08, 95% CI, 2.55,3.71), respectively, for daily cigarette smoking rather than not smoking cigarettes as compared who had not sexual intercourse. There is not much data on the precise effect of having several sex partners on male cigarette smoking. However, some research has linked cigarette smoking to a variety of sexual behaviors. For instance, one study indicated that smoking was linked to more sexual partners among men [31]. In a different study, it was discovered that young males who smoke cigarettes and have sex with men report having more casual and transactional partners than non-smokers [32]. According to a third study, having female partners is one of the risk variables for current frequent smoking among Chinese males who engage in male sex [33].

Men in the highest wealth index group were more likely to smoke than men in the lowest wealth index category. This result contrasts with research conducted in Ethiopia [15], five South Asian nations [24], Hungary [34], Ghana [23]. In general, those from lower socioeconomic class have less educational degrees and are more likely to be dependent on alcohol and tobacco. They are also less knowledgeable about the risks associated with smoking [35]. Wealth does not always result in a decrease in cigarette use [36]. Regarding other non-communicable diseases risk variables, investigations conducted in Egypt have revealed a pattern that is similar. In two of these studies, it was found that education protected against obesity and diabetes whereas wealth was found to increase obesity due to higher consumption of high-energy foods [35–38]. As we’ve already mentioned, our research revealed that males with greater money are more likely to smoke cigarettes. The study’s foundation is multinomial regression, which was used to base it on the frequency of cigarette smoking. Because of this, even while poor men may have a higher likelihood of smoking, they often lack the funds to do so. Other research examined participants’ smoking status or history, which may have contributed to the conclusion that poor men are more likely to smoke than their counterparts. Further research should concentrate on the wealth index to obtain a comprehensive explanation of the link between cigarette smoking and India’s high wealth index.

Similar to earlier research [24, 39, 40], we discovered that males who were employed smoked more cigarettes than men who were not employed. Working people, especially men, may encounter work stress, which may in turn have a favorable effect on smoking. Similarly, individuals can easily become smokers if they had unhealthy interactions with their coworkers or had come across smokers. It is commonly known that men’s smoking intensity and job stress are correlated [15, 41].

In the pooled analysis, we discovered that education significantly influenced cigarette smoking in east Africa while controlling for other characteristics. When compared to males who are typically uneducated, our final model’s results showed that men with a secondary or higher degree of education were less likely to smoke cigarettes. For instance, better education served as a barrier to smoking in a study carried out in five South Asian nations [24], including Malaysia [22], Sri Lanka [39], and Ethiopia [15]. This can be explained by the fact that education helps people become more aware of the negative effects of smoking on their health. More educated persons might use the national stop line and other support programs for quitting smoking. Higher educated individuals in India attempted to quit smoking more than less educated individuals [42].

It was discovered that rural residence has a negative statistically significant impact on smoking. Compared to men from urban origins, men from rural areas were less likely to consume cigarettes every day. Urban residents may be exposed to smoking environments more frequently, which alters people’s smoking habits under these circumstances. The tobacco business may easily target urban residents since marketing is more readily available in metropolitan areas. Urban residents are more prone to smoke cigarettes, according to a European study [24, 43].

Likely, men who had mass media exposure about cigarette smoking had shown a lower relative risk ratio of to be daily smoker by (RRR = 0.76, 95% CI, 0.73,0.81) as compared to unexposed individuals. Men’s cigarette smoking can be significantly impacted by exposure to the media. Adult smoking behavior can be altered by mass media campaigns, according to studies [44, 45]. Strong anti-tobacco mass media efforts and visual health warnings can deter youngsters and other vulnerable populations from starting to smoke, as well as boost the number of current smokers who give up [46]. When campaigns are a component of broader tobacco control initiatives, their effects on smoking cessation are more significant [45]. Reach, intensity, duration, and message type are all factors that influence the outcome of mass media campaigns [45]. A body of research supporting the notion that entertainment media can affect young people’s smoking habit has been conducted to examine the relationship between smoking in entertainment media and youth smoking [47].

### Strength and limitations of the study

The primary study strength is that it used a sizable sample size from nationally representative surveys with high response rates, excellent interviewer training, standardized data collection methods across countries, and consistent content across time which produced enough power to analyze the relationship between socio-demographic characteristics and cigarette smoking. The comparison of results across countries was made more powerful statistically by combining databases from ten different countries using pooled analysis. Furthermore, the accuracy and generalizability of the study are improved because the data were adequately sampled across the nation, with a high response rate. In addition, the study used a best fitted multinomial modeling technique to determine the frequencies of cigarette smoking. As a result, the current findings could be extrapolated to healthcare settings and socio-demographic features that are similar. However, this study’s disadvantage is that because the data were obtained from secondary sources and the surveys were cross-sectional, no conclusions about causality could be formed. Another cultural and perception-related variable across countries was not included in the study. Additionally, the self-reporting data was based on actual events, which could lead to recall bias. As a result of societal shame and people’s tendency to be conservative, the prevalence of cigarette smoking may be underreported.

## Conclusions

According to our data, men in East African countries were much more likely to smoke cigarettes if they were older, had less education, had a higher wealth index, were divorced/widowed, engaged in many sexual relationships, had early sexual activity, lived in urban region, were employed, or had no media exposures. To decrease smoking initiation and increase smoking cessation among men, policymakers and public health practitioners should consider the factors identified. An improvement in smoking cessation support services can also be an effective intervention.

Targeted education and awareness campaigns, smoke-free policies in urban areas, workplace smoking cessation programs, media-based anti-smoking campaigns, and longitudinal monitoring and surveillance are among the top policy implications of this study. However, it is important to consider the cultural and socio-economic context of East African countries while implementing these policy implications. Tailoring interventions to specific populations and addressing social determinants influencing smoking behavior will increase the likelihood of success in reducing smoking rates among men in East Africa.

## Data Availability

All data concerning this study are accommodated and presented in this document. The detailed data set can be freely accessible from the www.dhsprogram.com website.
